# Habitat fragmentation causes immediate and time-delayed biodiversity loss at different trophic levels

**DOI:** 10.1111/j.1461-0248.2010.01457.x

**Published:** 2010-05

**Authors:** Jochen Krauss, Riccardo Bommarco, Moisès Guardiola, Risto K Heikkinen, Aveliina Helm, Mikko Kuussaari, Regina Lindborg, Erik Öckinger, Meelis Pärtel, Joan Pino, Juha Pöyry, Katja M Raatikainen, Anu Sang, Constantí Stefanescu, Tiit Teder, Martin Zobel, Ingolf Steffan-Dewenter

**Affiliations:** 1 Population Ecology Group, Department of Animal Ecology I, University of Bayreuth, Universitätsstrasse 30D-95447 Bayreuth, Germany; 2 Department of Ecology, Swedish University of Agricultural SciencesP.O. Box 7044, SE-75007 Uppsala, Sweden; 3 CREAF (Center for Ecological Research and Forestry Applications), Autonomous University of BarcelonaE-08193 Bellaterra, Spain; 4 Finnish Environment Institute, Research Programme for BiodiversityP.O. Box 140, FI-00251 Helsinki, Finland; 5 Institute of Ecology and Earth Sciences, University of TartuLai 40, Tartu 511005, Tartu, Estonia; 6 Department of Systems Ecology, Stockholm UniversitySE-106 91 Stockholm, Sweden; 7 Butterfly Monitoring Scheme, Museu de Granollers de Ciències Naturals, Francesc Macià51, E-08402 Granollers, Spain

**Keywords:** Conservation, extinction cascades, extinction debt, grassland communities, habitat loss, habitat management, landscape change, relaxation time, species longevity

## Abstract

Intensification or abandonment of agricultural land use has led to a severe decline of semi-natural habitats across Europe. This can cause immediate loss of species but also time-delayed extinctions, known as the extinction debt. In a pan-European study of 147 fragmented grassland remnants, we found differences in the extinction debt of species from different trophic levels. Present-day species richness of long-lived vascular plant specialists was better explained by past than current landscape patterns, indicating an extinction debt. In contrast, short-lived butterfly specialists showed no evidence for an extinction debt at a time scale of c. 40 years. Our results indicate that management strategies maintaining the *status quo* of fragmented habitats are insufficient, as time-delayed extinctions and associated co-extinctions will lead to further biodiversity loss in the future.

## Introduction

Loss of biodiversity is a worldwide concern. One primary cause of species loss is habitat destruction and fragmentation (Tilman *et al.* 2001), but the rate of extinctions might be accelerated due to other causes such as invasion by alien species, overexploitation, climate change, habitat deterioration and extinction cascades (Diamond 1989; Thomas *et al.* 2004a; Brook *et al.* 2008; Dunn *et al.* 2009). Extinction processes often occur with a time delay and populations living close to their extinction threshold might survive for long time periods before they go extinct (Brooks *et al.* 1999; Hanski & Ovaskainen 2002; Lindborg & Eriksson 2004; Helm *et al.* 2006; Vellend *et al.* 2006). This time delay in extinction is called the ‘relaxation time’ (Diamond 1972) and the phenomenon that declining populations will eventually go extinct in fragmented or degraded habitats has been described as an ‘extinction debt’ (Tilman *et al.* 1994; Kuussaari *et al.* 2009). In present-day fragmented and perturbed landscapes, populations of many species might be on a deterministic path to extinction even without any further habitat loss occurring.

However, our understanding of the occurrence and ubiquity of extinction debts across ecosystems and taxonomic groups is highly incomplete and neither temporal nor spatial scales at which extinction debts occur are well known (Cousins 2009; Kuussaari *et al.* 2009). Regional studies have focused on a single taxonomic group (vascular plants or vertebrates) and their results have been contradictory, with some studies reporting evidence for the existence of an extinction debt (Brooks *et al.* 1999; Lindborg & Eriksson 2004; Helm *et al.* 2006), but others not (Adriaens *et al.* 2006). Further, little is known about the relevance of species traits such as longevity, resource specialistation or trophic rank in the context of delayed colonizations and extinctions as a result of environmental change (Menendez *et al.* 2006; Kuussaari *et al.* 2009; Jackson & Sax 2010).

**Figure 1:**
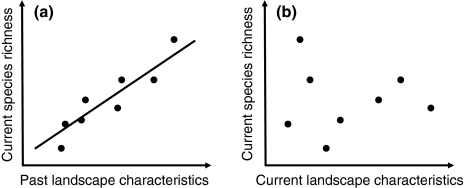
A concept for detecting extinction debt: past landscape characteristics explain current species richness better than current landscape characteristics.

Evidence for an extinction debt can be assumed when past landscape characteristics explain current species richness better than current landscape characteristics (Fig. 1; Kuussaari *et al.* 2009). Extinction debt might be expected to occur in recently fragmented semi-natural grasslands in Europe. Such grasslands represent regional biodiversity hotspots, with very high numbers of endangered plant and butterfly species (WallisDeVries *et al.* 2002; Cremene *et al.* 2005). Grassland habitats are globally threatened due to conversion into arable or urban land and the cessation of traditional extensive grazing regimes in recent decades (Sala *et al.* 2000; WallisDeVries *et al.* 2002; Hoekstra *et al.* 2005). European semi-natural grasslands are assumed to have lost 90% and in some regions even more of their former area during the last century (WallisDeVries *et al.* 2002). Due to such a drastic loss, the grasslands are likely to suffer from deterministic long-term decline of species specialized on these habitats, potentially resulting in an extinction debt. If extinction debts are occurring in these grasslands, management strategies aimed at maintaining only the *status quo* of the currently managed grasslands, need to be reconsidered urgently. Habitat area, connectivity and habitat quality would need to be improved to prevent future time-delayed extinctions (Margules & Pressey 2000; Lindenmayer *et al.* 2008; Hahs *et al.* 2009).

Vascular plants as primary producers, and butterflies as herbivores in the larval stage and as potential pollinators in the adult stage, have ecological key functions in grasslands and can be considered as conservation indicators for terrestrial habitats (WallisDeVries *et al.* 2002; Thomas *et al.* 2004b; Bloch *et al.* 2006). The two species groups differ in their individual longevity, with most grassland plant species being long-lived (perennial), whereas butterfly species are short-lived (van Groenendael *et al.* 1997; Nowicki *et al.* 2005). Long-lived species are expected to have a higher probability of showing time-delayed extinctions compared with short-lived species (Morris *et al.* 2008).

Our analyses provide the first large-scale evidence for future biodiversity loss due to the extinction debt for vascular plants, while butterfly species potentially have paid their debt with fast occurring extinctions after habitat perturbation. We conclude that the future loss of vascular plant species will inevitably lead to co-extinctions of specialized herbivores.

## Materials and methods

### Study region and sites

**Figure 2:**
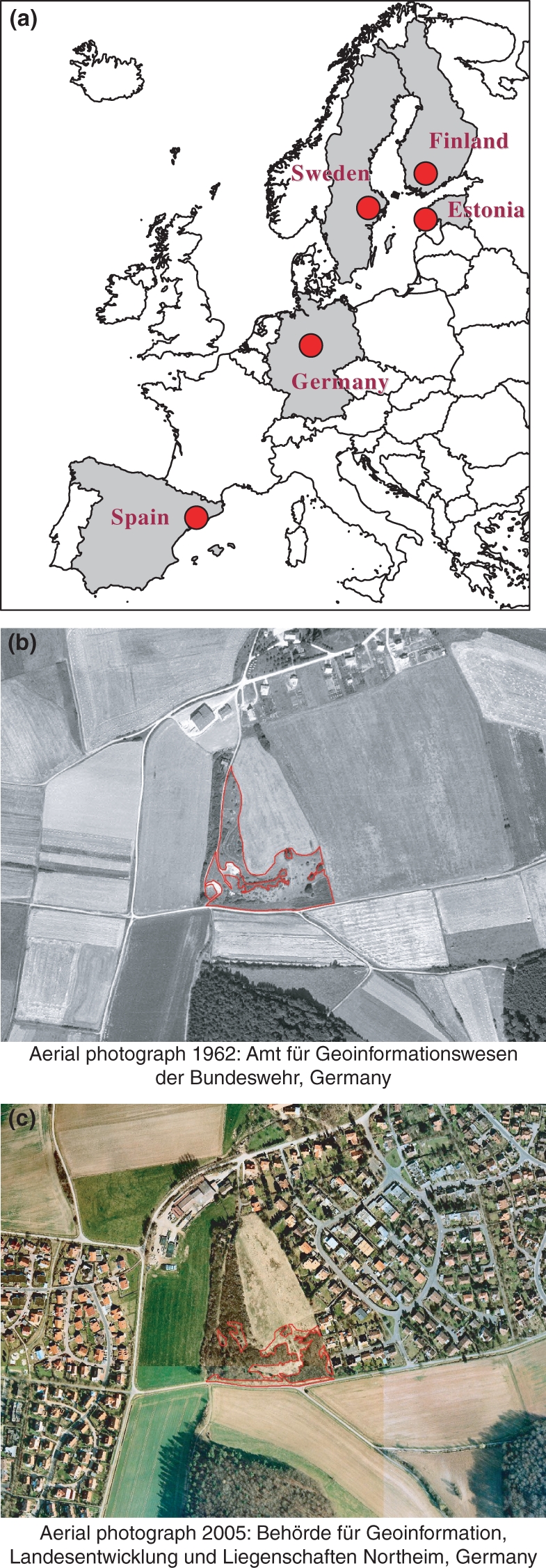
Study regions and land cover change. (a) Five European study regions (red circles), in which a total of 147 semi-natural grasslands were surveyed. (b, c) Habitat loss of calcareous grasslands and landscape changes are common throughout Europe. The study site example (outlined in red) shows a calcareous grassland patch in the German study region (b) in 1962 and (c) in 2005.

A total of 147 semi-natural grasslands were studied in five European countries (Estonia: 26 grassland patches, Finland: 30, Germany: 31, Spain: 30 and Sweden: 30) (Fig. 2a). Within each country species-rich semi-natural grasslands were selected, but the type of grasslands differed among countries. In Estonia, all studied grassland sites belonged to calcareous alvar grasslands, in Finland and Sweden to dry-mesic grasslands, in Germany to calcareous grasslands and in Spain to calcareous sub-mediterranean pastures. All the chosen semi-natural grassland types are fragmented and occur as discrete habitat patches with measurable patch area. Areas, which were strongly overgrown with bushes or trees were interpreted as non-grassland area in all study regions. The studied grassland types cover only a small percentage of area in the respective countries (e.g. in the German study region 0.26%) (Krauss *et al.* 2003), depend on regular management by grazing or mowing and represent regional and continental biodiversity hotspots in Europe (WallisDeVries *et al.* 2002; Cremene *et al.* 2005).

### Landscape data

Within each country, the studied grassland patches were chosen to cover a patch area gradient, although the gradients differ in range and mean patch area between countries (Table S1). A second criterion in choosing study sites was patch connectivity, and sites were selected to cover a gradient from more isolated to more connected sites. In order to measure connectivity, we quantified the area covered by the same grassland type within a 2-km buffer, including the study patch. The 2-km radius was chosen to reflect the potential average dispersal rates of vascular plant and butterfly species in the grasslands as estimated from previous studies (Moilanen & Nieminen 2002; Adriaens *et al.* 2006). The measure of landscape level habitat area is called here ‘landscape area’. Other types of connectivity measures, such as Hanski’s connectivity index, could not appropriately be used, as the high proportion of focal habitat in some areas, such as at some Estonian sites, did not allow meaningful calculations of connectivity indices or the use of distance to next habitat patch (Winfree *et al.* 2005). Nevertheless, different types of connectivity measures are highly correlated and the choice of measure is unlikely to affect the generality of our results (Moilanen & Nieminen 2002; Krauss *et al.* 2003; Winfree *et al.* 2005).

**Figure 3:**
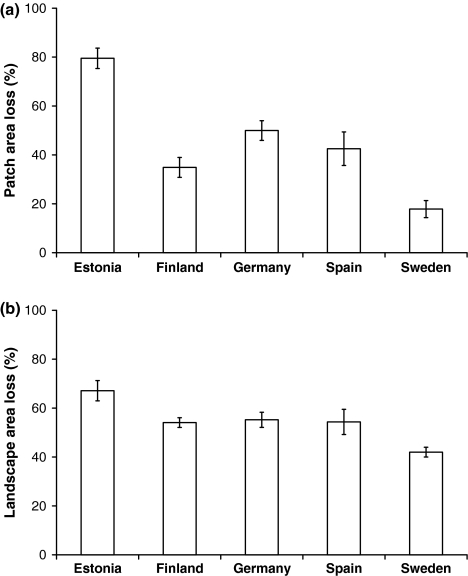
Loss of semi-natural grasslands in the five study countries. (a) patch area loss in percentage (habitat loss of the focal study site) and (b) landscape area loss in percentage (habitat loss in a 2 km buffer radius). Mean ± SE.

In order to estimate patch area and landscape area, we interpreted digital and orthorectified photographs of the study patches with a 2-km radius around the centre of each study patch. Aerial photographs were taken between 1999 and 2007 and were used to interpret the current grassland distribution in each country. Historical aerial photographs, mainly from the 1950s to 1960s from different sources, were used to quantify past explanatory variables. The time frame was for most study sites between 36 and 49 years, depending on country and availability of photographs (for details see Appendix S1). Digitalization, orthorectification and interpretation of the photographs were conducted by the company GISAT, Czech Republic (http://www.gisat.cz/) with additional background information including biotope mapping and expert advice from local field workers. Changes in area and connectivity of the grassland patches over the last five decades were quantified by examining historical and recent aerial photographs (Fig. 2b,c). Decline in patch area and loss of habitat within the 2-km landscape circles (landscape area loss) were calculated as the proportion of current to past areas (Fig. 3, Table S1).

### Biodiversity data

To investigate the existence of extinction debts in European semi-natural grasslands and across two trophic levels, we gathered a comprehensive quantitative data set of species richness of vascular plants and butterflies (including burnet moths) with extensive plot and transect surveys (for details see Appendix S1). In all the 147 study sites, vascular plant and butterfly species richness were recorded in one study year between 2000 and 2007 (a single year per country). All plant and butterfly species recorded from the study sites were classified separately for each country as a grassland specialist (i.e. a species-dependent or clearly favouring the focal grassland type) or as a generalist (a species not depending on the focal grassland type), with the help of field guides and local expert advice. Habitat-specialized species as well as the generalist species cover the whole gradient from rare to very abundant species. Only the grassland specialists were used in the subsequent statistical analyses, as non-specialists were not expected to be restricted to grassland patches, or logically linked with the measured connectivity values. Vascular plant species richness per patch was recorded in all five countries using study plots, and complemented by additional searching within study patches. Butterfly species richness always included burnet moths and was estimated based on three to seven transect walks in one study year (a constant number of walks in each country), when conditions were suitable for butterfly activity (for details see Appendix S1).

### Statistical analyses

The statistical analyses were performed using the software R 2.8.0 for Windows (R Development Core Team 2007). The two response variables in the analyses were species richness of (1) grassland plant specialists and (2) grassland butterfly specialists. The explanatory variables in the general linear mixed effects models were: (1) current patch area, (2) current landscape area, (3) past patch area and (4) past landscape area. To linearize relationships, explanatory variables were always log 10-transformed, whereas untransformed response variables met the assumptions of normality and homoscedasticity. Landscape area was measured as the amount of grassland habitat within a 2-km circle around the centre of each study patch, including the focal patch area. In all the regression models in which species richness was related to the four explanatory variables, country was included as a random intercept. We did not include random slopes *a priori* in the models, as slopes within countries should be similar and negative slopes in past species-area or landscape-area relationships would violate an extinction debt assumption. However, due to the essentially better model fits and the occurrence of negative or non-significant slopes (e.g. in Estonia and Finland), we also present the random slope and intercept models, as well as the relationships for each explanatory variable separately for each country in the Supporting Information (Figures S1 and S2, Tables S5 and S6). As all explanatory variables were correlated, we used Akaike Information Criterion for small sample sizes AICc (library bbmle in R), to determine the relative importance of the explanatory variables (Burnham & Anderson 2002). Using a multi-model study setting, we examined the AICc values for all the 15 possible models with all different combinations of the four explanatory variables. The overall importance of a given explanatory variable predicting richness patterns of plants and butterflies was measured by calculating the Akaike weights for each model compared with the full set of all models. In a second step, the Akaike weights for all models containing each explanatory variable separately were summed up to get the AICc sums, which measure the relative importance of each explanatory variable (Johnson & Omland 2004). If past habitat variables are more important for current species richness than current habitat variables, an extinction debt can be assumed (Kuussaari *et al.* 2009). To visualize the relative importance of the explanatory variables (current and past patch area, and current and past landscape area), we present partial residual figures (Zuur *et al.* 2007) taking into account the three other explanatory variables and country as a random intercept.

## Results

On an average, 18–80% of the previous patch area was lost per study region with a range between 0 to 99.8% area loss per habitat patch (Fig. 3, Table S1). On average historical habitat patches are the largest in Estonia (208 ± 29 ha). They also showed the highest average losses of 80%, whereas the other four countries with average patch areas of 2–11 ha had considerably lower losses (18–50%). Especially in Sweden, the patch area loss of the selected sites was relatively small (mean 18%, median 10%) (Fig. 3, Table S1). On a 2-km landscape scale, the loss of area was similar in all countries (42–67%). Current and past patch and current and past landscape area were in most countries correlated (Table S2). Current species richness of habitat-specialized vascular plants and butterflies decreased significantly with increasing patch area loss and increasing landscape area loss (see Appendix S1).

In total, we documented the occurrence of 872 plant and 140 butterfly species in the 147 grassland patches. Approximately half of these species were grassland specialists (404 plant and 76 butterfly species) (Tables S3 and S4). Only these specialized species were included in the statistical analyses to avoid bias due to invasive or ubiquitous plant species and migratory or generalist butterfly species. Indeed, statistical models for non-specialized species indicated no extinction debt (see Appendix S1).

**Figure 4:**
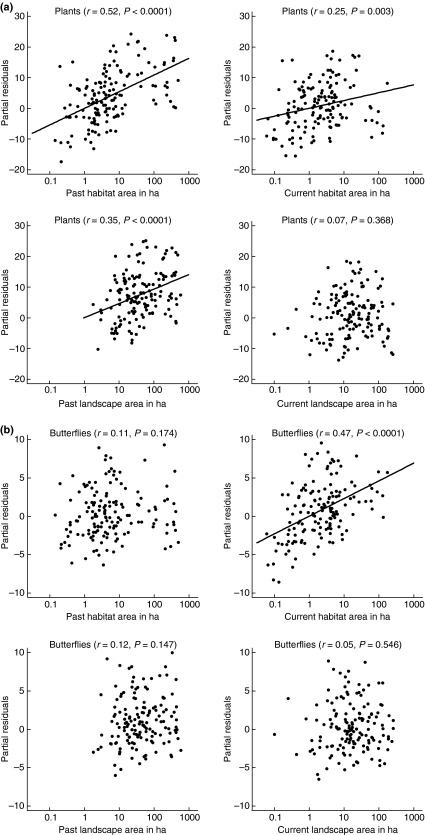
Evidence for extinction debt: importance of past vs. current grassland area for species richness of (a) specialized vascular plants and (b) specialized butterflies in five European countries. Partial residuals from the models in relation to past and current patch area, and past and current landscape area are shown with ‘country’ as a random effect to visualize the independent importance of the focal explanatory variable in the model. Regression lines are only shown when *P* < 0.05, *r* and *P* values of the partial regressions are presented in order to illustrate the figures (statistical AICc approach see Table 1).

We found that habitat-specialized vascular plants, but not butterflies, showed an extinction debt over a time frame of 36–49 years of rapid habitat loss. Past patch area and past landscape area both explained current species richness of plants across countries, even when species richness was corrected for all other explanatory variables (Fig. 4a). The weighted AICc values from models for plants ranked past patch area as the most important predictor for current species richness (AICc sum = 0.968) compared with the other explanatory variables (current patch area or current and past landscape area: AICc sums 0.543–0.580; Table 1). In models also including random slopes, the past landscape area was the best predictor for plant species richness, which also indicates the importance of past predictors for current species richness (Table S5a). However, considering each country separately, only grasslands in Germany showed a weak indication for an extinction debt for plants (Figure S1, Table S6a). This suggests that regional studies, e.g. at a country level, are likely to provide more limited chances to detect extinction debts than cross-country comparisons due to (1) a lower number of replicates compared with all study sites across countries, (2) correlations between explanatory variables within countries (Table S2) and (3) partly small habitat losses in some regions (Table S1).

**Table 1:** Importance of past and current explanatory variables in predicting species richness of plants and butterflies

Past patch area	Current patch area	Past landscape area	Current landscape area	*K*	AICc	Δ AICc	Likelihood	Akaike weight
*Plants*								
**X**	X	X		6	1037.58	0.00	1.00	0.258
**X**			X	5	1037.73	0.14	0.93	0.240
**X**	X		X	6	1039.04	1.46	0.48	0.124
**X**		X		5	1039.43	1.84	0.40	0.103
**X**		X	X	6	1039.49	1.90	0.39	0.100
**X**	X	X	X	7	1039.70	2.11	0.35	0.090
**X**	X			5	1041.36	3.78	0.15	0.039
	X	X		5	1042.60	5.02	0.08	0.021
**X**				4	1043.24	5.66	0.06	0.015
	X	X	X	6	1044.09	6.50	0.04	0.010
	X		X	5	1049.45	11.87	< 0.01	0.001
	X			4	1050.73	13.15	< 0.01	< 0.001
		X		4	1059.90	22.32	< 0.01	< 0.001
		X	X	5	1060.00	22.42	< 0.01	< 0.001
			X	4	1060.71	23.13	< 0.01	< 0.001
**0.968**	0.543	0.580	0.564		AICc sum			
*Butterflies*								
	**X**			4	782.38	0.00	1.00	0.276
	**X**	X		5	783.01	0.63	0.73	0.201
	**X**		X	5	783.65	1.27	0.53	0.146
X	**X**			5	783.80	1.43	0.49	0.135
X	**X**	X		6	784.99	2.61	0.27	0.075
X	**X**		X	6	785.12	2.74	0.25	0.070
	**X**	X	X	6	785.19	2.81	0.25	0.068
X	**X**	X	X	7	787.15	4.77	0.09	0.025
X			X	5	792.62	10.24	0.01	0.002
X		X	X	6	793.44	11.06	< 0.01	0.001
X				4	796.21	13.83	< 0.01	< 0.001
X		X		5	797.35	14.98	< 0.01	< 0.001
			X	4	800.54	18.16	< 0.01	< 0.001
		X	X	5	802.68	20.30	< 0.01	< 0.001
		X		4	805.99	23.61	< 0.01	< 0.001
0.309	**0.997**	0.370	0.312		AICc sum			

Bold letters indicates the most important explanatory variable.

Country was included in all models as a random factor.

Plants: full model: 5.43 (***past patch area***) + 2.55 (*current patch area*) + 4.68 (*past landscape area*) + 0.87 (*current landscape area*) + 40.02. Only the slope of ***past patch area*** is significantly different from zero.

Butterflies: full model: 0.43 (*past patch area*) + 2.31 (***current patch area***) + 0.67 *(past landscape area*) + 0.25 (*current landscape area*) + 7.31. Only the slope of ***current patch area*** is significantly different from zero.

*K*, Number of parameters; Likelihood, likelihood of the model being the best model.

X = included in the corresponding AICc model.

For butterflies, in contrast to plants, the current patch area was the best predictor of current species richness. Current patch area occurred in the eight best ranked AICc models (AICc sum = 0.997). Past explanatory variables and current landscape area were much less important (AICc sums 0.309–0.370; Table 1) and showed no relationship with butterfly species richness after taking into account the effects of all other explanatory variables (Fig. 4b). In addition, in models with random slopes, current patch area remained the best explanatory variable for butterflies (Table S5b). Testing each country separately confirmed that current patch area predicts current butterfly species richness best in four countries, whereas in Sweden current landscape area was the best predictor (Figure S2, Table S6b).

## Discussion

Our data indicate the existence of an extinction debt for plant specialists in European semi-natural grasslands. Consequently, an unknown proportion of the current plant diversity in this habitat type will go extinct if no new conservation actions aimed at large-scale habitat restoration are initiated. In contrast, butterflies responded to habitat perturbation on a shorter time scale and have probably paid most of their extinction debt. This is consistent with the expectation that short-lived species show short relaxation times and pay a possible extinction debt quickly (Kuussaari *et al.* 2009). For example, in Britain, butterflies have experienced more severe declines in recent decades, compared with long-lived vascular plants and birds (Thomas *et al.* 2004b). This also suggests that butterflies and other short-lived organisms respond more rapidly to environmental changes (Morris *et al.* 2008), and thus constitute better early warning indicators of fragmentation effects on biodiversity than other species groups.

Alternative explanations for the observed differences in extinction debt are possible. Habitat loss and fragmentation are known to affect trophic networks with high trophic levels being more susceptible to fragmentation than low trophic levels (Didham *et al.* 1998; Gilbert *et al.* 1998; Komonen *et al.* 2000). Reasons for the stronger response in higher trophic levels might be linked with lower population sizes, higher population variability and strong dependence on the lower trophic level. However, the population sizes between modular plants and unitary butterflies are difficult to compare, whereas it is plausible that population variability is higher for short-lived butterflies compared with long-lived plants, and it is evident that butterflies depend on larval host plants, adult nectar plants and roosting places during their life cycles (Dennis *et al.* 2003). Butterflies also have the possibility to disperse actively while plant dispersal is often passive. It is assumed that both species groups have similar dispersal rates in semi-natural grasslands (Moilanen & Nieminen 2002; Adriaens *et al.* 2006), but active dispersers might be able to recognize suitable breeding habitats and thus leave unsuitable habitats fast, while plants need to cope with habitat conditions until extinction.

To maintain plant diversity it is necessary to increase awareness of potential extinction debts, which might have remained unnoticed. If extinction debts are common in habitats which have recently been reduced in size, previous studies might have underestimated the future negative effects of habitat loss on species richness. A potential reason why some previous studies have not found any evidence for an extinction debt for plants (e.g. Adriaens *et al.* 2006) might be difficulties in determining the most relevant spatial or temporal scale of assessment, including the time-frame of initiation of severe habitat destruction and the amount of remaining habitat in the landscape (Cousins 2009). Apart from the possible sampling problems, these reasons might also explain why we found no clear indications for an extinction debt on a country level. In Finland and Sweden, the sharpest declines in grasslands were clearly before 1950, whereas it was after 1950 in Germany and Spain, and 1930–1970 in Estonia (see details in Appendix S1). Across countries, our data indicate that the analysed time scale of *c.* 40 years with an average of 18–80% focal patch area loss in the five study regions are appropriate scales for detecting an extinction debt of long-lived plants in temperate grasslands.

Although our data set is hitherto the largest available testing for the existence of extinction debt and the only one where responses of plant and animal taxa have been compared on the same sites, a number of questions regarding extinction debts remain open. Most importantly, our data did not allow calculating the magnitude of extinction debt underlining the urgent need for standardized monitoring schemes to obtain long-term time series for functionally important species groups (Kuussaari *et al.* 2009). Nonetheless our analyses revealed that habitat specialized vascular plants in grasslands show an extinction debt across Europe. Time lags in extinction were also predicted for native vegetation in modern cities across the globe (Hahs *et al.* 2009). We conclude that counteractions to protect biodiversity of long-living plant specialists are still possible and urgently required. If these actions are not undertaken in the highly fragmented semi-natural grasslands across Europe, then not only vascular plants will go extinct, but cascading trophic effects would inevitably lead to co-extinctions of associated specialist herbivore species, including further grassland butterfly species, and specialized parasitoids at higher trophic levels (Koh *et al.* 2004; Brook *et al.* 2008; Dunn *et al.* 2009; Haddad *et al.* 2009). Our data also demonstrate rapid responses of butterflies to habitat loss. Importantly, habitat loss takes place not only because of habitat destruction but is increasingly caused by changing climatic conditions (Thomas *et al.* 2004a). Thus, novel and large-scale conservation measures have to be implemented now to prevent a rapid loss of diversity in the future.

## SUPPORTING INFORMATION

**Figure S1** Effects of past and current patch area and past and current landscape area on specialized vascular plant species richness in five European countries (regression lines are only shown, when significantly positive (*P* < 0.05); best country model in box, see Table S6a).

**Figure S2** Effects of past and current patch area and past and current landscape area on specialized butterfly species richness in five European countries (regression lines are only shown, when significantly positive (*P* < 0.05); best country model in box, see Table S6b).

**Table S1** Habitat and landscape characteristics of semi-natural grasslands in five European countries.

**Table S2** Pearson correlations between the response variables of specialized vascular plant species richness and specialized butterfly species richness and between the explanatory variables in the five study countries.

**Table S3** Grassland specialized plant species recorded in this study.

**Table S4** Grassland specialized butterfly species recorded in this study.

**Table S5** Importance of past and current explanatory variables in predicting species richness of (a) plants and (b) butterflies with country and slope of current patch area as random factors.

**Table S6** AICc sums for each country and each explanatory variable separately for (a) plants and (b) butterflies.

**Appendix S1** Supporting text (study sites in different countries; biodiversity surveys in different countries; additional analyses; references).

As a service to our authors and readers, this journal provides supporting information supplied by the authors. Such materials are peer-reviewed and may be re-organized for online delivery, but are not copy-edited or typeset. Technical support issues arising from supporting information (other than missing files) should be addressed to the authors.
