# Supplementary material for: Habitat fragmentation causes immediate and time-delayed biodiversity loss at different trophic levels
Source: Ecol Lett. 2010 May;13(5):597–605. doi: 10.1111/j.1461-0248.2010.01457.x (PMC2871172; doi:10.1111/j.1461-0248.2010.01457.x)
Supplement: Supplementary file 1 [file ele0013-0597-SD1.pdf]

Supporting Information

Appendix S1. Supporting text (Study sites in different countries; Biodiversity surveys in different countries; Additional analyses; References)

Study sites in different countries.

All aerial photographs have been interpreted by the company GISAT, Czech Republic (http://www.gisat.cz/) with additional background information including biotope mappings and the help of local experts and field workers. The historical aerial photographs were often only available as analog black and white printouts, which have been digitalized and orthorectified by GISAT, if not otherwise mentioned in the country information.

Estonia: In Estonia, 26 dry calcareous alvar grasslands were chosen on the islands of Saaremaa and Muhu, west of Estonian mainland. Current patch and landscape areas were obtained from color digital orthophotos with GSD of 50 cm (scale 1:10000). Photos were taken by Estonian Land Board in 2005. The historical aerial photographs from 1968 originate from archives of Estonian Land Board. Scale of photos is approximately 1:16000. Photographs were digitalized in Estonia. There is a time period of 37 years between current and historical landscape information.

Habitat area and configuration in 1930s is believed to correspond to state of alvar grasslands that has persisted for centuries (Laasimer 1965; Pärtel *et al*. 1999; Helm *et al*. 2006). Habitat loss and fragmentation in Estonian alvar grasslands started after 1930s and by 1968 around half of the original habitat area was lost.

Finland: The Finnish study sites (n = 30) were situated in two regions, Uusimaa (n = 20) and Pirkanmaa (n = 10) in southwestern Finland (Raatikainen *et al*. 2007; Pöyry *et al*. 2009). In

the study year of 2000, 17 of the sites were managed as pastures, whereas 13 of the sites had been abandoned from grazing 2-35 years prior to the study year and were subject to gradual overgrowth. However, only sites not covered by forest were included in the study. The current patch and landscape areas were obtained by interpretation of digitalized and orthorectified color aerial photographs (scale: 1:30000) taken between 1999 and 2005. The past patch and landscape areas were obtained by interpretation of digitalized and orthorectified black and white aerial photographs (scale 1:60000, scanning resolution 15 µm) taken in the year 1963 (except one area that was photographed in 1965).

In SW Finland (covering all the Finnish study areas) decline in the occurrence of open semi-natural habitats started already around 1880, and according to agricultural land-use statistics the sharpest decline was passed in these areas already by 1920 (Pöyry *et al*. 2004). Following this early decline, the amount of semi-natural habitats has continued to decrease steadily until these days (Luoto *et al*. 2003) although the relative rate of decline might have been lower during the recent decades than it was in the late 1800s and early 1900s.

Germany: In Germany, in the vicinity of the city of Göttingen in Lower Saxony, 31 calcareous grassland patches were surveyed (Krauss *et al*. 2003; 2004). The current patch and landscape areas were achieved by interpretation of digitalized and orthorectified color aerial photographs. The current aerial photographs from 2004 and 2005 had a resolution of 20 – 40 cm on a scale of 1:12000 and were acquired from „Behörde für Geoinformation, Landesentwicklung und Liegenschaften Northeim“, „Hessisches Landesamt für Bodenmanagment und Geoinformation“ and „Landesamt für Vermessung und Geoinformation Freistaat Thüringen“. The vast majority of the historical black and white photos is from 1962, had a resolution of 50 cm on a scale of 1:18000 and was achieved from “Amt für Geoinformationswesen der Bundeswehr”. Further photographs were acquired from „Landesvermessungs und Geobasisinformation Niedersachsen“ from the years 1967 – 1968

on a scale of 1:12000 or 1:13000, from „Hessisches Landesamt für Bodenmanagment und Geoinformation“ from the year 1959 and from ”Landesvermessung und Geobasisinformation Brandenburg” from the year 1971. Approximately 90% of the 31 patches were interpreted for 1962 and 2005, which is a time period of 43 years between current and historic landscape variables.

The maximum distribution of calcareous grasslands in the region is assumed to be in the 19th century with the most essential losses in area being in the 20th century (Von Drachenfels personal communication). In southern Germany there is evidence for a severe habitat loss of calcareous grassland after 1960, with a reduction of more than 50 % until 1990. Between 1900 and 1960 the habitat loss in this region was only 25 % (WallisDeVries *et al*. 2002).

Spain: In Spain, 30 calcareous submediterranean pastures dominated by hemicryptophytes and chamaephytes (Al. *Aphyllanthion monspeliensis*) were selected in the calcareous massifs in Southern Catalonia (NE of Spain, 41ºN, 0º 30’ E). These habitat patches were scattered across 100 km, distributed in 4 dry mountain platforms sited at more than 800 m a.s.l. and showing a mountain Mediterranean climate with cold winters and mild summers (average temperature, 14.1ºC, average annual rainfall 650 mm). Patches summarize a variety of sizes and intensities of change (from stable to high regression), in contrastingly forested landscapes. The historical (1956) aerial photographs were obtained from archives of Spanish Army. Scale of original photos was approximately 1:30000, and pixel size of the resulting orthophoto-maps was 1m. Present-day (2004) images corresponded to orthophoto-maps assembled and orthorectified by the Cartographic Institute of Catalonia (www.icc.cat). Thus, there is a time period of 48 years between current and historical landscape information. Both historical and present-day grassland patches of the study areas were digitized by the Spanish team, by on-screen photo-interpretation of the respective orthophotomaps.

There are no general data for the study sites about the onset and maximum rate of

pasture loss, but in general it is assumed that it started in the decade of the 1940, in accordance with the observed in nearby regions such as Southern France (Debussche *et al*. 1999). Historical data, only available for some municipalities in the study region, confirm this trend (Lloret *et al*. 2002).

Sweden: 30 dry to mesic semi-natural grasslands situated in the counties of Östergötland and Uppsala, south-east Sweden, were selected. Current and historical patch and landscape areas were obtained by interpretation of orthorectified aerial photographs The current aerial photographs were digital photos taken between 1999 and 2005 (the majority in 2003) from a height of 4800 m and had a resolution of 0.5 m. The historical aerial photographs were digitized (scanning resolution 15 mµ) black and white analog photos taken from a height of 4600 m in 1952 -1960 (the majority in 1956-59) and had a resolution of 0.5 m. Both current and historical photographs were acquired from the National Land Survey of Sweden (Lantmäteriet).

The number of semi-natural grasslands has decreased drastically during the last century. Today only 10% of the semi-natural grassland and 2% of the hay meadows remain. The largest decrease took place before 1950 and during the last decades the decrease has slowed down mostly due to restoration of former semi-natural grasslands (Eriksson *et al*. 2002).

Biodiversity surveys in different countries. Plant species richness in Estonia was recorded in July 2001 with one intensive survey in a 30 m radius circle area per patch (see details in Helm *et al*. 2006). Such sampling covers the total number of specialist species from the whole habitat patch due to the homogeneity of alvar grasslands, confirmed by additional transect walks over remaining areas. In Finland one survey was conducted between June and August 2000 using one 0.25 ha plot and 15 x 1 m^2 plots, plus a searching time adjusted to habitat area to complement the species list (for details see Raatikainen *et al*. 2007). In Germany one

survey in May/June plus one in August 2000 were conducted. Depending on patch area 1 – 3 plots with 25 m² area were mapped and additional plant transects per patch in the same year to complete species richness data (details see Krauss *et al*. 2004). In Spain and Sweden plant records were conducted in 2007. One survey in March and one in May with 25 x 0.5 m² plots plus additional transects were conducted in Spanish grasslands. In Sweden one survey in July with 10 x 1 m² plus additional transects per patch were carried out.

Butterfly transects (including burnet moths) were carried out in 2000 in Finland and Germany, and in 2007 in Estonia, Spain and Sweden. All transects were adjusted to patch area and conducted between end of April and end of August to cover the whole flying period of butterfly species. In Estonia four transects, in Finland seven, in Germany five, in Spain three and in Sweden four transects were conducted under conditions suitable for butterfly activity (Pollard 1977; for details on Finnish and German transects see Krauss *et al*. 2003; Pöyry *et al*. 2009).

Additional analyses

In addition to the extinction debt analyses, we tested whether (1) patch area loss and (2) landscape area loss of each study site (N = 147) explains current species richness of plants and butterflies. Species richness of specialized vascular plants (patch area loss: slope -7.61, p = 0.007; landscape area loss: slope -8.30, p = 0.03) and butterflies (patch area loss: slope -3.23, p = 0.005; landscape area loss: slope -4.81, p = 0.003) decreased significantly with increasing area loss. Country was included as a random intercept in these general linear mixed effects models.

To validate the findings for the habitat specialists, we tested whether habitat generalists also showed an indication for an extinction debt. We assumed they should not, as their distribution is not restricted to semi-natural grasslands. The calculations indeed showed

no indication for an extinction debt, as slopes for full models, as shown for specialists in Table 1, were only significant for current patch area (generalist vascular plants: slope 5.69, p = 0.023; generalist butterflies: slope 1.94, p = 0.038), but not for past or landscape explanatory variables.

References

Debussche, M., Lepart, J. & Dervieux, A. (1999). Mediterranean landscape changes: evidence from old postcards. *Global Ecol. Biogeogr.*, 8, 3-15.

Eriksson, O., Cousins, S.A.O. & Bruun, H.H. (2002). Land-use history and fragmentation of traditionally managed grasslands in Scandinavia. *J. Veg. Sc.*, 13, 743-748.

Helm, A., Hanski, I. & Pärtel, M. (2006). Slow response of plant species richness to habitat loss and fragmentation. *Ecol. Lett.*, 9, 72-77.

Karsholt, O., Razowski, J. (1996). *The Lepidoptera of Europe. A distributional checklist* Apollo Books, Stenstrup.

Krauss, J., Klein, A.-M., Steffan-Dewenter, I. & Tscharntke, T. (2004). Effects of habitat area, isolation, and landscape diversity on plant species richness of calcareous grasslands. *Biodiv. Conserv.*, 13, 1427-1439.

Krauss, J., Steffan-Dewenter, I. & Tscharntke, T. (2003). How does landscape context contribute to effects of habitat fragmentation on diversity and population density of butterflies? *J. Biogeogr.*, 30, 889-900.

Laasimer, L. (1965). *Eesti NSV taimkate*. (Valgus, Tallinn).

Lloret, F.; Calvo, E.; Pons, X. & Díaz-Delgado, R. (2002). Wildfires and landscape patterns in Eastern Iberian Peninsula. *Landsc. Ecol.*, 17, 745-759.

Luoto, M., Rekolainen, S., Aakkula, J. & Pykälä, J. (2003). Loss of plant species richness and habitat connectivity in grasslands associated with agricultural change in Finland. *Ambio*

32:447-452.

Pollard, E. (1977). A method for assessing changes in the abundance of butterflies. *Biol. Conserv.*, 12, 115-134.

Pärtel, M., Mandla, R. & Zobel, M. (1999). Landscape history of a calcareous (alvar) grassland in Hanila, western Estonia, during the last three hundred years. *Landsc. Ecol.*, 14, 187-196.

Pöyry, J., Heliölä, J., Ryttäri, T. & Alanen, A. (2004). Perinnebiotooppien lajiston uhanalaistuminen. In: *Elämää pellossa. Suomen maatalousympäristön monimuotoisuus* (eds Tiainen, J., Kuussaari, M., Laurila, I.P. & Toivonen, T.). Edita, Helsinki, Finland, pp. 220-233.

Pöyry, J., Paukkunen, J., Heliölä, J. & Kuussaari, M. (2009). Relative contributions of local and regional factors to species richness and total density of butterflies and moths in semi-natural grasslands. *Oecologia,* 160, 577-587.

Raatikainen, K.M., Heikkinen, R.K. & Pykälä, J. (2007). Impacts of local and regional factors on vegetation of boreal semi-natural grasslands. *Plant Ecol.,* 189, 155-173.

WallisDeVries, M.F., Poschlod, P. & Willems, J.H. (2002). Challenges for the conservation of calcareous grasslands in northwestern Europe: integrating the requirements of flora and fauna. *Biol. Conserv.,* 104, 265-273.

Figure S1. Effects of past and current patch area and past and current landscape area on specialized vascular plant species richness in five European countries (regression lines are only shown, when significantly positive ($p < 0.05$); best country model in box, see Table S6a).

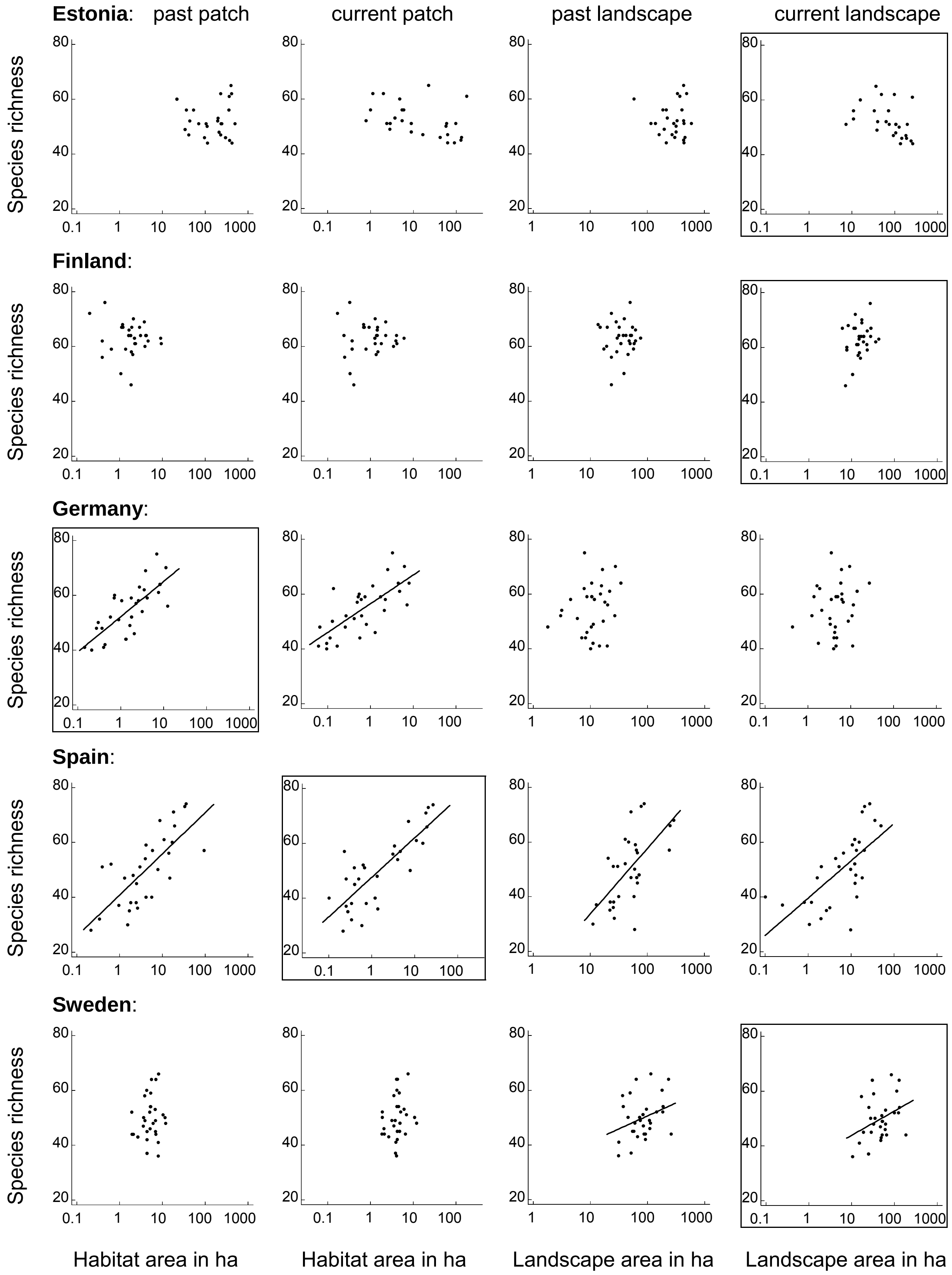

Figure S2. Effects of past and current patch area and past and current landscape area on specialized butterfly species richness in five European countries (regression lines are only shown, when significantly positive ($p < 0.05$); best country model in box, see Table S6b).

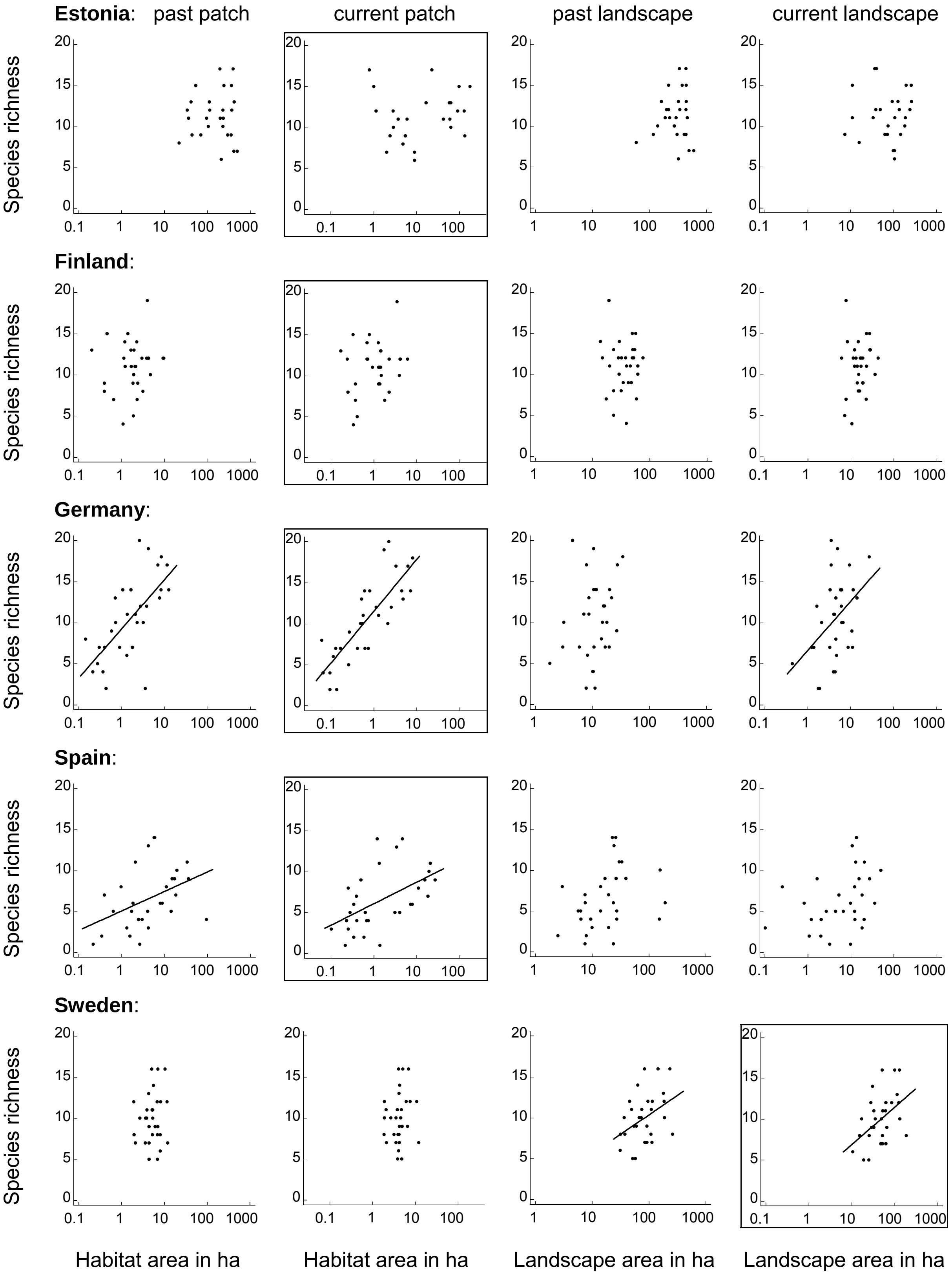

Table S1. Habitat and landscape characteristics of semi-natural grasslands in five European countries.

		Patch area loss (%)	Landscape area loss (%)	Current patch area (ha)	Past patch area (ha)	Current landscape area (ha)	Past landscape area (ha)
**Estonia**	Mean ± SE	79.5 ± 4.2	67.1 ± 4.1	38.91 ± 9.78	208.51 ± 28.50	103.81 ± 15.24	314.83 ± 25.74
N = 26	Median	86.2	70.0	8.97	205.5	95.4	321.8
	Range	20.9 – 99.6	31.3 – 95.1	0.78 – 176.70	22.03 – 495.40	7.33 – 261.70	58.60 – 590.30
**Finland**	Mean ± SE	34.9 ± 4.1	54.10 ± 2.0	1.56 ± 0.26	2.51 ± 0.40	17.55 ± 1.57	38.78 ± 2.96
N = 30	Median	32.9	55.5	1.28	1.91	15.61	38.21
	Range	0.0 – 94.3	31.1 – 73.4	0.17 – 6.09	0.20 – 9.51	6.09 – 42.93	13.63 – 76.08
**Germany**	Mean ± SE	50.0 ± 4.0	55.2 ± 3.1	1.67 ± 0.39	3.00 ± 0.61	5.93 ± 0.93	12.93 ± 1.36
N =31	Median	54.0	55.3	0.61	1.75	4.53	10.69
	Range	2.9 – 96.1	20.5 – 89.7	0.06 – 8.10	0.15 – 12.57	0.44 – 27.23	1.81 – 34.26
**Spain**	Mean ± SE	42.5 ± 6.9	54.3 ± 5.2	5.05 ± 1.37	10.67 ± 3.35	11.51 ± 2.08	32.15 ± 8.63
N = 30	Median	36.5	57.0	0.97	4.09	10.26	19.45
	Range	0.0 – 99.8	5.3 – 99.0	0.10 – 26.85	0.21 – 94.66	0.10 – 49.88	2.49 – 191.76
**Sweden**	Mean ± SE	17.8 ± 3.5	42.0 ± 2.0	4.60 ± 0.43	5.75 ± 0.47	59.11 ± 7.69	97.19 ± 10.89
N = 30	Median	9.9	33.4	4.19	5.34	49.90	84.45
	Range	0.0 – 60.1	26.7 – 67.4	1.85 – 11.96	1.93 – 12.03	10.47 – 187.27	31.10 – 262.71

Table S2. Pearson correlations between the response variables of specialized vascular plant species richness and specialized butterfly species richness and between the explanatory variables in the five study countries.

		Plant species richness	Past patch area	Current landscape area	Past landscape area
**Estonia**	Butterfly species richness	0.133 ns			
N = 26	Current patch area		0.356 (*)	0.751 ***	0.306 ns
	Past patch area			0.513 **	0.735 ***
	Current landscape area				0.598 ***
**Finland**	Butterfly species richness	0.574 ***			
N = 30	Current patch area		0.808 ***	0.347 (*)	0.333 (*)
	Past patch area			0.178 ns	0.296 ns
	Current landscape area				0.861 ***
**Germany**	Butterfly species richness	0.612 ***			
N = 31	Current patch area		0.890 ***	0.327 (*)	0.187 ns
	Past patch area			0.285 ns	0.206 ns
	Current landscape area				0.810 ***
**Spain**	Butterfly species richness	0.512 **			
N = 30	Current patch area		0.627 ***	0.632 ***	0.411 *
	Past patch area			0.412 *	0.600 ***
	Current landscape area				0.738 ***
**Sweden**	Butterfly species richness	0.697 ***			
N = 30	Current patch area		0.833 ***	-0.131 ns	-0.178 ns
	Past patch area			-0.297 ns	-0.272 ns
	Current landscape area				0.966 ***

(*** p < 0.001; ** p < 0.01; * p < 0.05; (*) p < 0.1, ns p > 0.1)

Table S3. Grassland specialized plant species recorded in this study

Plant species	Estonia	Finland	Germany	Spain	Sweden
Achillea millefolium	X	X	X		X
Achillea ptarmica					X
Acinos arvensis	X		X		
Agrimonia eupatoria	X		X		X
Agrostis capillaris	X	X			X
Agrostis vinealis	X				
Ajuga genevensis			X		
Ajuga pyramidalis		X			X
Alchemilla glabra					X
Alchemilla glaucescens		X			X
Alchemilla monticola					X
Alchemilla sp.		X			
Allium oleraceum		X			
Allium schoenoprasum			X		
Alopecurus pratensis		X			X
Althaea hirsuta				X	
Alyssum spinosum				X	
Anemone nemorosa		X			
Anemone sylvestris			X		
Angelica sylvestris		X			
Antennaria dioica	X	X	X		X
Anthemis tinctoria	X		X		
Anthericum liliago				X	
Anthoxanthum odoratum	X	X	X		X
Anthriscus sylvestris		X			
Anthyllis fontqueri				X	
Anthyllis montana				X	
Anthyllis vulneraria	X		X		
Aphyllanthes monspeliensis				X	
Arabidopsis thaliana		X			
Arabis auriculata				X	
Arabis glabra		X			
Arabis hirsuta	X		X		
Arctostaphylos uva-ursi				X	
Arenaria serpyllifolia		X			
Arenaria tetraqueta condensata				X	
Argyrolobium zannoni				X	
Aristolochia pistolochia				X	
Arnica montana					X
Arrhenatherum album				X	
Arrhenatherum elatius			X		
Arrhenatherum sardoum				X	
Artemisia campestris	X				
Artemisia rupestris	X				
Asperula cynanchica				X	
Asperula tinctoria	X				
Asphodelus cerasiferus				X	
Asterolinon linum-stellatum				X	
Astragalus danicus	X				
Astragalus glycyphyllos			X		
Astragalus monspessulanus				X	

monspessulanus					
Avenula pratensis iberica				X	
Bistorta vivipara		X			X
Botrychium lunaria		X			X
Botrychium multifidum		X			
Brachypodium pinnatum	X	X	X		
Brimeura amethystina fontqueri				X	
Briza media	X		X		X
Bromus erectus			X		
Bromus hordeaceus		X	X		
Bupleurum fruticescens				X	
Bupleurum rigidum				X	
Calamagrostis epigejos		X			
Calluna vulgaris	X				
Campanula glomerata	X	X	X		
Campanula patula		X			
Campanula persicifolia	X	X	X		X
Campanula rapunculoides			X		X
Campanula rotundifolia	X	X	X		X
Cardamine pratensis					X
Cardaminopsis arenosa			X		
Carduncellus monspelliensium				X	
Carduus nigrescens nigrescens				X	
Carduus nutans			X		
Carex caryophyllea	X		X		
Carex ericetorum	X				
Carex flacca	X		X		
Carex halleriana				X	
Carex hirta					X
Carex humilis				X	
Carex leporina			X		
Carex muricata		X			
Carex ornithopoda	X		X		
Carex ovalis		X			X
Carex pallescens		X			X
Carex panicea		X			
Carex pilulifera		X			X
Carex spicata		X			X
Carex tomentosa	X				
Carlina vulgaris	X		X		X
Carum carvi		X			X
Catananche coerulea				X	
Centaurea jacea	X	X	X		X
Centaurea linifolia caballeroi				X	
Centaurea linifolia linifolia				X	
Centaurea montana lingulata				X	
Centaurea phrygia		X			
Centaurea scabiosa			X		X
Centaurium erythraea			X		
Centaurium quadrifolium barrelieri				X	
Cerastium arvense			X		
Cerastium fontanum	X	X			X
Cerastium gracile				X	
Cerastium holosteoides			X		

Cerastium pumilum				X	
Cerastium semidecandrum	X				
Cerinthe minor			X		
Cirsium acaule	X		X		
Cirsium heterophyllum		X			
Cirsium tuberosum			X		
Cirsium vulgare		X			
Cistus clusii				X	
Clinopodium vulgare			X		
Conopodium majus ramosum				X	
Convolvulus arvensis			X		
Convolvulus lanuginosus				X	
Coris monspeliensis				X	
Coronilla minima				X	
Crepis tectorum	X				
Crucianella angustifolia				X	
Crupina vulgaris				X	
Cuscuta epithymum				X	
Cynosurus cristatus					X
Dactylis glomerata	X	X	X		X
Dactylorhiza incarnata					X
Dactylorhiza maculata					X
Danthonia decumbens	X				X
Daucus carota	X		X		X
Deschampsia cespitosa		X			X
Deschampsia flexuosa					X
Dianthus deltoides	X	X			X
Dianthus pungens tarraconensis				X	
Dictamnus hispanicus				X	
Dipcadi serotinum				X	
Dorycnium pentaphyllum pentaphyllum				X	
Echinaria capitata				X	
Echinops ritro				X	
Echinops sphaerocephalus			X		
Echium vulgare	X		X		
Elytrigia repens			X		
Erica multiflora				X	
Erigeron acer	X				
Erinacea anthyllis				X	
Erophila verna		X			X
Erysimum grandiflorum				X	
Euphorbia cyparissias			X		
Euphorbia flavicoma mariolensis				X	
Euphorbia nicaeensis nicaeensis				X	
Euphrasia nemorosa		X			X
Euphrasia officinalis			X		
Euphrasia stricta		X			
Falcaria vulgaris			X		
Festuca nigrescens				X	
Festuca ovina	X	X	X	X	X
Festuca paniculata spadicea				X	
Festuca pratensis	X	X	X		X
Festuca rubra	X	X	X		X
Filipendula vulgaris	X		X		X

Fragaria vesca	X	X			X
Fragaria viridis	X		X		
Fritillaria pyrenaica				X	
Fumana ericoides				X	
Fumana procumbens				X	
Fumana thymifolia				X	
Galium boreale	X	X			
Galium mollugo	X		X		X
Galium parisiense				X	
Galium pinetorum				X	
Galium pumilum			X		
Galium saxatile					X
Galium uliginosum		X			
Galium verum	X	X	X		X
Genista hispanica				X	
Genista scorpius				X	
Genista tinctoria			X		
Gentianella campestris		X			X
Gentianella ciliata			X		
Gentianella germanica			X		
Geranium pusillum		X			
Geranium sylvaticum		X			
Geum rivale		X			X
Gladiolus illyricus				X	
Globularia vulgaris				X	
Gymnadenia conopsea	X	X	X	X	
Helianthemum appeninum				X	
Helianthemum marifolium marifolium				X	
Helianthemum nummularium	X		X	X	X
Helianthemum oelandicum italicum				X	
Helianthemum origanifolium molle				X	
Helichrysum stoechas				X	
Helictotrichon pratense	X				
Helictotrichon pubescens	X	X	X		X
Heracleum sibiricum		X			
Hieracium caespitosum			X		
Hieracium lachenalii			X		
Hieracium pilosella	X	X	X	X	X
Hieracium sp.		X			
Hieracium spp.		X			
Hieracium umbellatum	X	X			
Hippocrepis comosa			X	X	
Hypericum maculatum		X			X
Hypericum perforatum	X	X	X		X
Hypochoeris maculata		X			X
Hypochoeris radicata				X	
Iberis ciliata ciliata				X	
Inula conyza			X		
Inula montana				X	
Inula salicina	X				
Iris chamaeiris				X	
Juncus conglomeratus					X
Juniperus communis			X		
Knautia arvensis	X	X	X		X

Knautia subscaposa				X	
Koeleria pyramidata			X		
Koeleria vallesiana				X	
Lathyrus filiformis				X	
Lathyrus linifolius					X
Lathyrus pratensis	X	X			X
Lathyrus saxatilis				X	
Lavandula angustifolia				X	
Lavandula latifolia				X	
Leontodon autumnalis	X	X			
Leontodon hispidus	X	X	X		
Leucanthemum vulgare	X	X	X		X
Leuzea conifera				X	
Linaria vulgaris			X		
Linum catharticum	X		X		X
Linum narbonense				X	
Linum strictum strictum				X	
Linum tenuifolium suffruticosum				X	
Listera ovata			X		
Lithospermum fruticosum				X	
Lotus corniculatus	X		X		X
Luzula campestris	X	X			X
Luzula multiflora		X			
Luzula pallescens		X			
Lychnis viscaria		X			
Malva moschata			X		
Medicago lupulina	X		X		
Medicago minima				X	
Medicago sativa falcata			X		
Melampyrum arvense			X		
Melampyrum nemorosum			X		
Minuartia hybrida				X	
Myosotis ramosissima		X			X
Myosotis stricta		X			
Narcissus assoanus				X	
Nardus stricta		X			
Omalotheca sylvatica		X			
Onobrychis supina supina				X	
Onobrychis viciifolia			X		
Ononis minutissima				X	
Ononis pusilla				X	
Ononis repens			X		
Ononis spinosa			X		
Ophrys apifera			X		
Ophrys fusca				X	
Ophrys insectifera			X		
Orchidaceae				X	
Orchis mascula			X	X	
Orchis militaris	X		X		
Orchis purpurea			X		
Orchis tridentata			X		
Origanum vulgare			X		
Orobanche caryophyllea				X	
Orobanche sp				X	

Parnassia palustris		X			
Peucedanum officinale stenocarpum				X	
Phleum bertolonii	X				
Phleum phleoides	X				X
Phleum pratense	X	X			X
Phlomis lychnitis				X	
Picris hieracioides		X			
Pimpinella major					X
Pimpinella saxifraga	X	X	X		X
Plantago lanceolata	X	X	X		X
Plantago media	X		X		X
Platanthera bifolia		X			X
Platanthera chlorantha			X		X
Poa angustifolia	X	X			
Poa compressa	X	X			
Poa pratensis		X	X		X
Poa trivialis					X
Polygala calcarea				X	
Polygala comosa	X		X		
Polygala monspeliaca				X	
Polygala rupestris rupestris				X	
Polygala vulgaris	X		X		X
Potentilla anserina					X
Potentilla argentea		X			
Potentilla crantzii		X			
Potentilla erecta		X			
Potentilla neumanniana	X		X	X	
Potentilla reptans	X				X
Primula veris	X		X		X
Prunella grandiflora			X		
Prunella vulgaris	X	X	X		X
Ranunculus acris	X	X	X		X
Ranunculus auricomus		X			X
Ranunculus bulbosus	X		X		X
Ranunculus gramineus				X	
Ranunculus polyanthemos	X	X			
Rhamnus alaternus var balearicus				X	
Rhamnus saxatilis				X	
Rhinanthus angustifolius		X	X		
Rhinanthus minor	X	X	X		X
Rosa dumalis ssp. coriifolia		X			
Rosa dumalis ssp. dumalis		X			
Rosa rubiginosa			X		
Rosa sp.	X				
Rosa villosa		X			
Rosmarinus officinalis				X	
Rubus caesius	X				
Rumex acetosa		X			X
Rumex acetosella		X			
Sagina nodosa	X				
Salix starkeana		X			
Salvia officinalis lavandulifolia				X	
Salvia pratensis			X		
Sanguisorba minor			X	X	

Santolina chamaecyparissus				X	
Saponaria ocymoides				X	
Satureja montana				X	
Saxifraga granulata					X
Saxifraga tridactylites	X				
Scabiosa columbaria			X		
Scleranthus annuus		X			
Scorzonera angustifolia				X	
Scorzonera hirsuta				X	
Scorzonera hispanica crispatula				X	
Scorzonera humilis					X
Sedum acre	X	X			
Sedum album	X				
Sedum sediforme				X	
Sedum sexangulare			X		
Selinum carvifolia		X			
Senecio erucifolius			X		
Senecio jacobaea	X		X		
Serratula nudicaulis				X	
Serratula tinctoria					X
Seseli libanotis	X				
Seseli montanum montanum				X	
Sesleria caerulea	X				
Sideritis spinulosa ilicifolia				X	
Silene italica nevadensis				X	
Silene nutans			X		X
Silene vulgaris vulgaris			X		
Solidago virgaurea	X	X	X		
Stachys germanica			X		
Stachys heraclea				X	
Staehelina dubia				X	
Stellaria graminea		X			X
Stipa offneri				X	
Stipa pennata iberica				X	
Succisa pratensis	X	X			X
Taraxacum officinale	X				
Taraxacum spp.		X			
Teucrium botrys				X	
Teucrium polium				X	
Thalictrum tuberosum				X	
Thesium humifusum				X	
Thlaspi perfoliatum			X		
Thymelaea tinctoria				X	
Thymus serpyllum chamaedrys			X		
Thymus serpyllum fontqueri				X	
Thymus serpyllum serpyllum	X				X
Thymus vulgaris				X	
Tragopogon porrifolius australis				X	
Tragopogon pratensis					X
Trifolium arvense		X			X
Trifolium aureum		X			
Trifolium campestre			X		X
Trifolium dubium			X		
Trifolium medium		X	X		

Trifolium montanum	X		X		X
Trifolium ochroleucon			X		
Trifolium pratense	X	X	X		X
Trifolium repens	X	X			X
Trifolium spadiceum		X			
Trifolium stellatum				X	
Trigonella monspeliaca				X	
Trinia glauca				X	
Tulipa sylvestris australis				X	
Valeriana officinalis		X			
Valeriana tuberosa				X	
Verbascum lychnitis			X		
Verbascum thapsus		X			
Veronica arvensis		X			
Veronica austriaca subsp. teucrium			X		
Veronica austriaca tenuifolia				X	
Veronica chamaedrys	X	X	X		X
Veronica officinalis	X	X			X
Veronica serpyllifolia		X			X
Veronica spicata	X				X
Veronica verna		X			
Vicia cracca	X	X			X
Vicia hirsuta			X		
Vicia sepium		X			
Vicia tetrasperma		X			
Vincetoxicum hirundinaria	X		X		
Viola canina	X	X			X
Viola hirta	X		X		
Viola rupestris	X				
Viola tricolor		X			X
Xeranthemum inapertum				X	
**Species richness per country**	**106**	**126**	**130**	**141**	**105**
**Total species richness**			**404**		

X = occurred and categorized as specialized grassland species in the respective country

Table S4. Grassland specialized butterfly species recorded in this study

Butterfly species	Estonia	Finland	Germany	Spain	Sweden
Adscita statices		X			
Aphantopus hyperantus		X	X		X
Arethusana arethusa				X	
Argynnis adippe		X			X
Argynnis aglaja		X	X	X	X
Argynnis niobe	X	X		X	
Aricia agestis			X		
Aricia artaxerxes	X	X			X
Aricia eumedon	X	X			
Boloria euphrosyne			X		X
Boloria selene	X	X			X
Brenthis ino		X			
Carcharodus lavatherae				X	
Coenonympha arcania			X		X
Coenonympha glycerion	X	X			
Coenonympha hero	X				
Coenonympha pamphilus	X	X			X
Colias alfacariensis			X	X	
Colias hyale	X				
Cupido minimus	X		X	X	X
Cupido osiris				X	
Erebia medusa			X		
Erebia triaria				X	
Erynnis tages	X		X	X	X
Euphydryas aurinia	X		X	X	
Glaucopsyche alexis					X
Hamearis lucina	X				
Hesperia comma	X		X	X	X
Hipparchia fidia				X	
Hipparchia semele	X				
Hipparchia statilinus				X	
Hyponephele lycaon	X			X	
Lasiommata megera			X		X
Leptidea sinapis/reali	X		X		X
Lycaena alciphron				X	
Lycaena hippothoe	X	X			X
Lycaena phlaeas	X	X	X		X
Lycaena virgaureae	X	X			X
Maculinea arion	X				
Maniola jurtina					X
Melanargia galathea			X		
Melanargia occitanica				X	
Melitaea aurelia	X		X		
Melitaea cinxia	X			X	
Melitaea didyma				X	
Muschampia proto				X	
Papilio machaon			X		
Plebeius argus			X	X	
Polyommatus amandus	X	X			X
Polyommatus coridon			X	X	
Polyommatus daphnis				X	

Polyommatus escheri				X	
Polyommatus icarus		X			X
Polyommatus nivescens				X	
Polyommatus ripartii				X	
Polyommatus semiargus	X	X			X
Pseudophilotes baton				X	
Pyrgus alveus	X	X		X	
Pyrgus malvae	X	X			X
Pyrgus malvoides				X	
Satyrus actaea				X	
Scolitantides orion		X			
Spialia sertorius			X	X	
Thymelicus acteon			X	X	
Thymelicus lineola		X	X		
Thymelicus sylvestris				X	
Zygaena carniolica			X		
Zygaena filipendulae	X		X		X
Zygaena lavandulae				X	
Zygaena lonicerae			X		X
Zygaena minos	X				
Zygaena osterodensis					X
Zygaena purpuralis			X		
Zygaena sarpedon				X	
Zygaena transalpina			X		
Zygaena viciae		X	X		X
**Species richness per country**	**28**	**22**	**28**	**34**	**26**
**Total species richness**			**76**		

X = occurred and categorized as specialized grassland species in the respective country

Nomenclature follows (Karsholt & Razowski 1996).

Table S5. Importance of past and current explanatory variables in predicting species richness of (*A*) plants and (*B*) butterflies with country and slope of current patch area as random factors.

A Plants

past patch area	current patch area	**past landscape area**	current landscape area	K	AICc	Δ AICc	Likeli-hood	Akaike weight
X		**X**		6	1007.46	0	1	0.250
X	X	**X**		7	1008.70	1.24	0.54	0.134
		**X**		5	1008.95	1.49	0.47	0.118
	X	**X**		6	1009.20	1.74	0.42	0.104
X			X	6	1009.54	2.08	0.35	0.088
X		**X**	X	7	1009.66	2.20	0.33	0.083
		**X**	X	6	1010.92	3.46	0.18	0.044
X	X	**X**	X	8	1010.97	3.51	0.17	0.043
	X	**X**	X	7	1011.02	3.57	0.17	0.042
X	X		X	7	1011.37	3.91	.014	0.035
X				5	1011.64	4.19	0.12	0.031
X	X			6	1013.06	5.60	0.06	0.015
			X	5	1015.24	7.78	0.02	0.005
	X		X	6	1015.73	8.27	0.02	0.004
	X			5	1017.14	9.68	0.01	0.002
0.680	0.380	**0.819**	0.345	AICc sum				

Full model: 2.87 (*past patch area*) + 3.30 (*current patch area*) + 5.37 (***past landscape area***) - 0.08 (*current landscape area*) + 43.31
None of the slopes is significantly different from zero

B Butterflies

past patch area	**current patch area**	past landscape area	current landscape area	K	AICc	∆ AICc	Likeli-hood	Akaike weight
	**X**	X		6	773.60	0.00	1.00	0.202
	**X**		X	6	773.90	0.30	0.86	0.175
			X	5	775.24	1.64	0.44	0.089
	**X**	X	X	7	775.29	1.70	0.43	0.087
	**X**			5	775.57	1.97	0.37	0.076
X	**X**	X		7	775.61	2.01	0.37	0.074
		X		5	775.92	2.33	0.31	0.063
X	**X**		X	7	776.01	2.41	0.30	0.061
		X	X	6	776.89	3.29	0.19	0.039
X			X	6	777.01	3.41	0.18	0.037
X	**X**	X	X	8	777.53	3.94	0.14	0.028
X	**X**			6	777.69	4.09	0.13	0.026
X		X		6	778.07	4.48	0.11	0.022
X		X	X	7	779.04	5.44	0.07	0.013
X				5	780.19	6.59	0.04	0.008
0.269	**0.729**	0.530	0.529	AICc sum				

Full model: -0.14 (*past patch area*) + 2.44 (***current patch area***) + 1.31 (*past landscape area*) + 0.69 (*current landscape area*) + 6.71
Only the slope of ***current patch area*** is significantly different from zero

K = Number of parameters; Likelihood = Likelihood of the model being the best model

Bold letters = the most important explanatory variable

X = included in the corresponding AICc model

The table shows the AICc weights of each of the 15 possible models and the AICc sums for each explanatory variable separately for a, plants and b, butterflies. Species richness of the habitat specialists in five European countries is the response variable and current and past patch area and current and past landscape area are the explanatory variables in the models. The AICc sums show that species richness of plants is best predicted by past landscape area, while in butterflies current patch area is the most important explanatory variable.

Table S6. AICc sums for each country and each explanatory variable separately for (*A*) plants and (*B*) butterflies.

A Plants

<table>
<tr><th>past patch area</th><th>current patch area</th><th>past landscape area</th><th>current landscape area</th><th></th><th>Country</th></tr>
<tr><td>0.408</td><td>0.509</td><td>0.285</td><td>0.618</td><td>AIC Sum:</td><td>Estonia</td></tr>
<tr><td colspan="6">Full model: 4.41 (past patch area) -1.85 (current patch area) + 2.17(past landscape area) - 5.99 (current landscape area) + 50.36
None of the slopes is significantly different from zero</td></tr>
<tr><td>0.259</td><td>0.233</td><td>0.751</td><td>0.863</td><td>AIC Sum:</td><td>Finland</td></tr>
<tr><td colspan="6">Full model: -1.83 (past patch area) + 1.06 (current patch area) - 22.68 (past landscape area) + 26.50 (current landscape area) + 66.72
Only the slope of current landscape area is significantly different from zero</td></tr>
<tr><td>0.826</td><td>0.439</td><td>0.304</td><td>0.282</td><td>AIC Sum:</td><td>Germany</td></tr>
<tr><td colspan="6">Full model: 7.45 (past patch area) + 5.58 (current patch area) + 10.87 (past landscape area) – 8.79 (current landscape area) + 48.64
None of the slopes is significantly different from zero</td></tr>
<tr><td>0.646</td><td>0.988</td><td>0.707</td><td>0.348</td><td>AIC Sum:</td><td>Spain</td></tr>
<tr><td colspan="6">Full model: 5.43 (past patch area) + 8.93 (current patch area) + 5.33 (past landscape area) + 2.14 (current landscape area) + 36.92
Only the slope of current patch area is significantly different from zero</td></tr>
<tr><td>0.350</td><td>0.411</td><td>0.432</td><td>0.625</td><td>AIC Sum:</td><td>Sweden</td></tr>
<tr><td colspan="6">Full model: 7.23 (past patch area) + 3.20 (current patch area) - 13.14 (past landscape area) + 21.03 (current landscape area) + 32.72
None of the slopes is significantly different from zero</td></tr>
</table>

B Butterflies

past patch area	current patch area	past landscape area	current landscape area		Country
0.215	**0.389**	0.373	0.333	AIC Sum:	**Estonia**

Full model: -1.09 (*past patch area*) **+1.59 (*current patch area*)** + 4.86 (*past landscape area*) -2.86 (*current landscape area*) + 5.30
None of the slopes is significantly different from zero

0.334	**0.500**	0.370	0.385	AIC Sum:	**Finland**

Full model: 0.26 (*past patch area*) **+ 1.67 (*current patch area*)** – 8.28 (*past landscape area*) + 7.47 (*current landscape area*) + 14.77
None of the slopes is significantly different from zero

0.437	**1.000**	0.265	0.551	AIC Sum:	**Germany**

Full model: -2.75 (*past patch area*) **+ 7.83 (*current patch area*)** – 1.84 (*past landscape area*) + 3.82 (*current landscape area*) + 11.79
Only the slope of ***current patch area*** is significantly different from zero

0.303	**0.836**	0.256	0.255	AIC Sum:	**Spain**

Full model: 0.41 (*past patch area*) **+ 2.24 (*current patch area*)** + 0.98 (*past landscape area*) – 0.15 (*current landscape area*) + 4.80
None of the slopes is significantly different from zero

0.521	0.304	0.465	**0.833**	AIC Sum:	**Sweden**

Full model: 8.98 (*past patch area*) – 4.94 (*current patch area*) - 13.99 (*past landscape area*) **+ 17.05 (*current landscape area*)** + 4.90
Only the slope of ***current landscape area*** is significantly different from zero

Bold letters = the most important explanatory variable

Species richness of the habitat specialists in five European countries is the response variable and current and past patch area and current and past landscape area are the explanatory variables in the models.
